# Porcine milk exosomes modulate the immune functions of CD14^+^ monocytes in vitro

**DOI:** 10.1038/s41598-023-48376-y

**Published:** 2023-12-05

**Authors:** Gabriela Ávila Morales, Daria De Leonardis, Joel Filipe, Rafaela Furioso Ferreira, Alessandro Agazzi, Helga Sauerwein, Marcello Comi, Vladimir Mrljak, Cristina Lecchi, Fabrizio Ceciliani

**Affiliations:** 1 Department of Veterinary Medicine and Animal Sciences, Università Degli Studi di Milano, Lodi, Italy; 2 Institute of Animal Science, Physiology and Hygiene Unit, University of Bonn, Bonn, Germany; 3 Faculty of Veterinary Medicine, University of Zagreb, Zagreb, Croatia; 4 Department of Veterinary Science for Health, Animal Production and Alimentary Security, Università Degli Studi di Milano, Lodi, Italy; 5 Department of Human Science and Quality of Life Promotion, Università Telematica San Raffaele, Rome, Italy

**Keywords:** Monocytes and macrophages, Cell biology

## Abstract

Exosomes mediate near and long-distance intercellular communication by transferring their molecular cargo to recipient cells, altering their biological response. Milk exosomes (MEx) are internalized by immune cells and exert immunomodulatory functions in vitro. Porcine MEx can accumulate in the small intestine, rich in macrophages. No information is available on the immunomodulatory ability of porcine MEx on porcine monocytes, which are known precursors of gut macrophages. Therefore, this study aims at (1) assessing the in vitro uptake of porcine MEx by porcine monocytes (CD14+), and (2) evaluating the in vitro impact of porcine MEx on porcine monocytes immune functions. MEx were purified by ultracentrifugation and size exclusion chromatography. The monocytes’ internalization of PKH26-labeled MEx was examined using fluorescence microscopy. Monocytes were incubated with increasing exosome concentrations and their apoptosis and viability were measured. Lastly, the ability of MEx to modulate the cells’ immune activities was evaluated by measuring monocytes’ phagocytosis, the capacity of killing bacteria, chemotaxis, and reactive oxygen species (ROS) production. MEx were internalized by porcine monocytes in vitro. They also decreased their chemotaxis and phagocytosis, and increased ROS production. Altogether, this study provides insights into the role that MEx might play in pigs’ immunity by demonstrating that MEx are internalized by porcine monocytes in vitro and exert immunomodulatory effects on inflammatory functions.

## Introduction

Exosomes are nano-sized extracellular vesicles (30–200 nm) with an endosome-derived limiting membrane that mediate intercellular communication in physiological and pathological conditions^1–3^. They are produced by all cell types, including immune cells, through the inward budding of the endosomal membrane, which mature into multivesicular bodies (MVB) that when fusing with the plasma membrane release the exosomes through exocytosis^4^.

As mediators of cell-to-cell communication, exosomes are secreted from donor cells to modulate short and long-distance signalling events by carrying and transferring their cargo, which can include proteins, both transmembrane and cytosolic proteins, lipids, DNA, RNA (mRNA, miRNA, non-coding RNA) and metabolites^1^. The exosomes’ cargo can then be transferred to recipient cells, altering their function^5, 6^. Several studies report that immune cell-derived exosomes can indeed functionally transfer miRNA after their fusion with the acceptor immune cells and modulate their gene expression^7, 8^.

Over the past two decades, accumulating evidence has reinforced the hypothesis that exosomes can induce, amplify and/or modulate both innate and adaptive immune responses, including natural killer cells activation, macrophage differentiation and monocyte chemotaxis induction^9^, antigen presentation, T cell activation and differentiation^10^. Immunosuppressive and anti-inflammatory roles of exosomes have also been described^11^. Indeed, mesenchymal stromal cells-derived exosomes exert immunomodulatory properties in human peripheral blood mononuclear cells (PBMC) and T cells, by increasing cell apoptosis, inducing the differentiation of T helper type 1 (Th1) into T helper type 2 (Th2), suppressing the secretion of the proinflammatory cytokines tumor necrosis factor alpha (TNF-α) and IL-1β, and increasing the production of the anti-inflammatory cytokine transforming growth factor beta (TGF-β)^12^.

Exosomes are present in different body fluids such as blood, saliva, urine, semen, cerebrospinal fluid, bile, and milk^5, 13^. Milk exosomes (MEx) are part of the complex mechanism of transmission of immunity from the mother to the offspring. By transferring immunoregulatory molecules, such as miRNA, exosomes may play an essential role in developing the newborn immune system and growth^14^. Indeed, human MEx resist digestion in vitro and are internalized by intestinal cells, affecting their gene expression^15^. Similarly, bovine MEx are internalized by human and rat intestinal cells where they release their miRNA cargo^16^.

Immune cells like human macrophages can also take up in vitro breast MEx, containing functional miRNA^17^. Inter-species exosome internalization has also been observed with bovine MEx, as they can enter humans^18^ and murine macrophages, and splenocytes in vitro^19^. Both, bovine and porcine MEx, including their cargo, can also accumulate in piglets and mice peripheral tissues rich in immune cells such as the liver, spleen, lung and the small intestine after suckling or oral administration^20^. Therefore, MEx could modulate the animals’ immunity after reaching the intestinal tract, as shown in vitro^21^. As further supported by proteomic analysis, bovine MEx proteins are mainly involved in immunological pathways such as Fc-gamma receptor-mediated phagocytosis, antigen processing and presentation, lymphocyte receptor signalling, and NK cell-mediated cytotoxicity^22^.

Porcine MEx have been shown to promote porcine intestinal epithelial cells proliferation in vitro^23^, and the production of intestinal secretory immunoglobulinA (SIgA) levels in piglets in vivo^24^. Moreover, porcine colostral exosomes increased in vitro the proportion of cytototxic T cells in PBMC from suckling piglets, suggesting a critical role in the developmemt of their cellular immunity^25^. Still, their effects on another important innate immune cell population such as porcine monocytes—known precursors of gut macrophages—have been so far unexplored. Therefore, this study aimed to investigate the in vitro effects of porcine MEx on some innate immune functions of monocytes, including viability, apoptosis, chemotaxis, oxidative burst, phagocytosis, and killing capacity.

## Results

### Purification and characterization of milk porcine exosomes

Nanoparticle tracking analysis (NTA) was performed to examine and determine the size distribution and concentration of the isolated LPS-depleted MEx (Supplementary Video 1, Supplementary Fig. 1A). According to NTA data, the mode size of the purified porcine MEx was 155 nm ± 8.9 nm (SEM) and the concentration was 2.55 × 10^10^ particles/mL (considering a dilution factor of 1:50).

The structural and morphological features of porcine LPS-depleted MEx were assessed by Transmission electron microscopy (TEM) negative staining. The exosomes presented a round or cup shape (Supplementary Fig. 1B) and were enclosed by a lipid bilayer membrane, which is more evident at higher magnifications (Supplementary Fig. 1C).

The protein content of the LPS-depleted MEx was quantified with the BCA protein quantification kit, and a total concentration of 147 μg/mL was obtained (data not shown). The presence of the exosome marker TSG-101 in MEx before and after LPS depletion was confirmed by Western blot analysis (Supplementary Fig. 1D). The detected band for this cytosolic protein was observed at the reported molecular weight (44 kDa).

### Characterization of exosomes immunomodulatory effects on porcine monocytes

#### Porcine monocytes internalized porcine MEx in vitro

**Figure 1:**
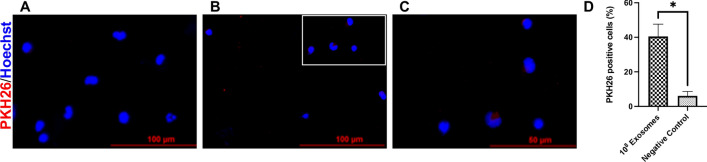
Uptake of LPS-depleted porcine milk exosomes (MEx) by porcine monocytes in vitro. Cells were incubated with 10^8^ of PKH26-labeled MEx or PKH26-PBS (negative control) for 22 h, their nuclei stained with Hoechst and examined by fluorescence microscopy: red, PKH26-labeled exosomes; blue, nuclei. Fluorescence microscopy image of cells treated with (**A**) PKH26-PBS; (**B**, **C**) PKH26-labeled MEx [(**C**) shows a higher magnification of the delineated area with white rectangle from (**B**)]; (**D**) Percentage of PKH26 positive cells that internalize PKH26-labeled exosomes. Data are means ± SEM of four experiments. Significance was declared at* P* ≤ 0.05 (*). Scale bars: 100 and 50 $\upmu$m, respectively.

To evaluate whether porcine monocytes can take up porcine MEx, the cells were co-cultured with 10^8^ PKH26-labeled MEx (LPS-depleted) for 22 h and then visualized using fluorescence microscopy. No fluorescence was observed in the PKH26-labeled PBS (negative control) treated cells (Fig. 1A). Porcine PKH26-labeled MEx (in red) were internalized by the monocytes (blue) in vitro, and they were mainly located in the cells’ cytoplasm, surrounding the nucleus as shown in Fig. 1B,C (which is a magnification of Fig. 1B). These results were further confirmed when determining the percentage of PKH26-positive cells, which was higher (*P* = 0.03) in the exosome group than in the negative control (Fig. 1D), which presented some background signal.

#### Porcine MEx did not affect porcine monocytes’ spontaneous apoptosis and viability

**Figure 2:**
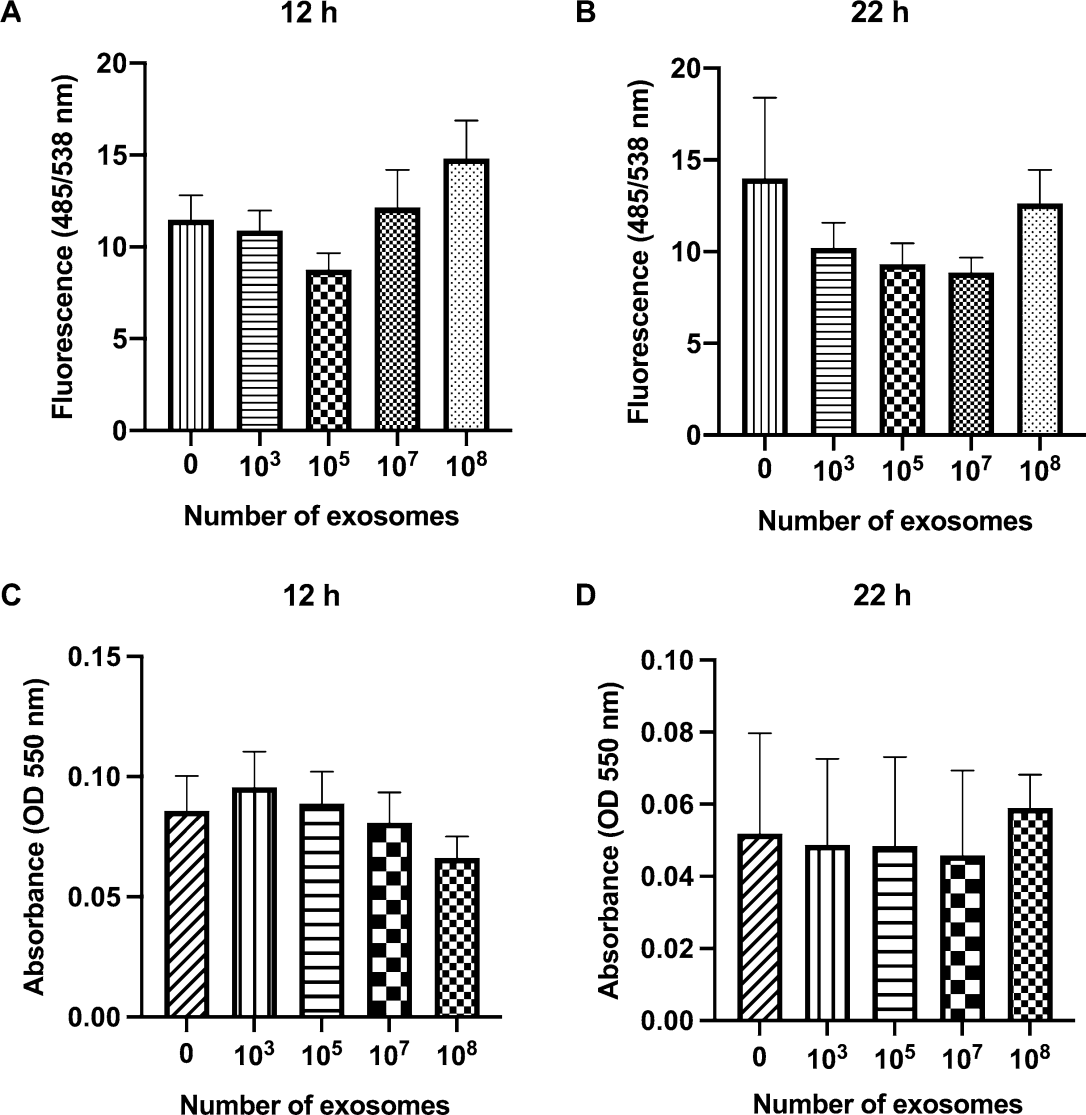
In vitro effect of LPS-depleted porcine milk exosomes (MEx) on porcine monocytes’ apoptosis and viability. Cells were treated with increasing numbers (10^3^,10^5^,10^7^ and 10^8^) of porcine MEx or only medium as control (no exosomes), and apoptosis at (**A**) 12 h and (**B**) 22 h was measured. Cells’ viability at (**C**) 12 h and (**D**) 22 h was also examined. Caspase-3/7 enzymatic activity and 2,5-diphenyl tetrazolium bromide (MTT) reduction by metabolically active cells were measured for apoptosis and viability, respectively. The results are expressed as fluorescence intensity (485/538 nm) for apoptosis and absorbance (OD 550 nm) for viability. Data are means ± SEM of five experiments. OD, optical density.

To determine if the MEx could exert toxic effects on porcine monocytes, cells were incubated with increasing numbers (10^3^, 10^5^, 10^7^, and 10^8^) of exosomes or with a complete medium only as control (no exosomes) for 12 and 22 h and their apoptosis and viability were measured. Increasing numbers of MEx showed no toxic effects on porcine monocytes during the time, as neither their spontaneous apoptosis nor viability at 12 h (Fig. 2A,C) and 22 h (Fig. 2B,D) were affected.

#### Porcine MEx decreased porcine monocytes’ chemotaxis

**Figure 3:**
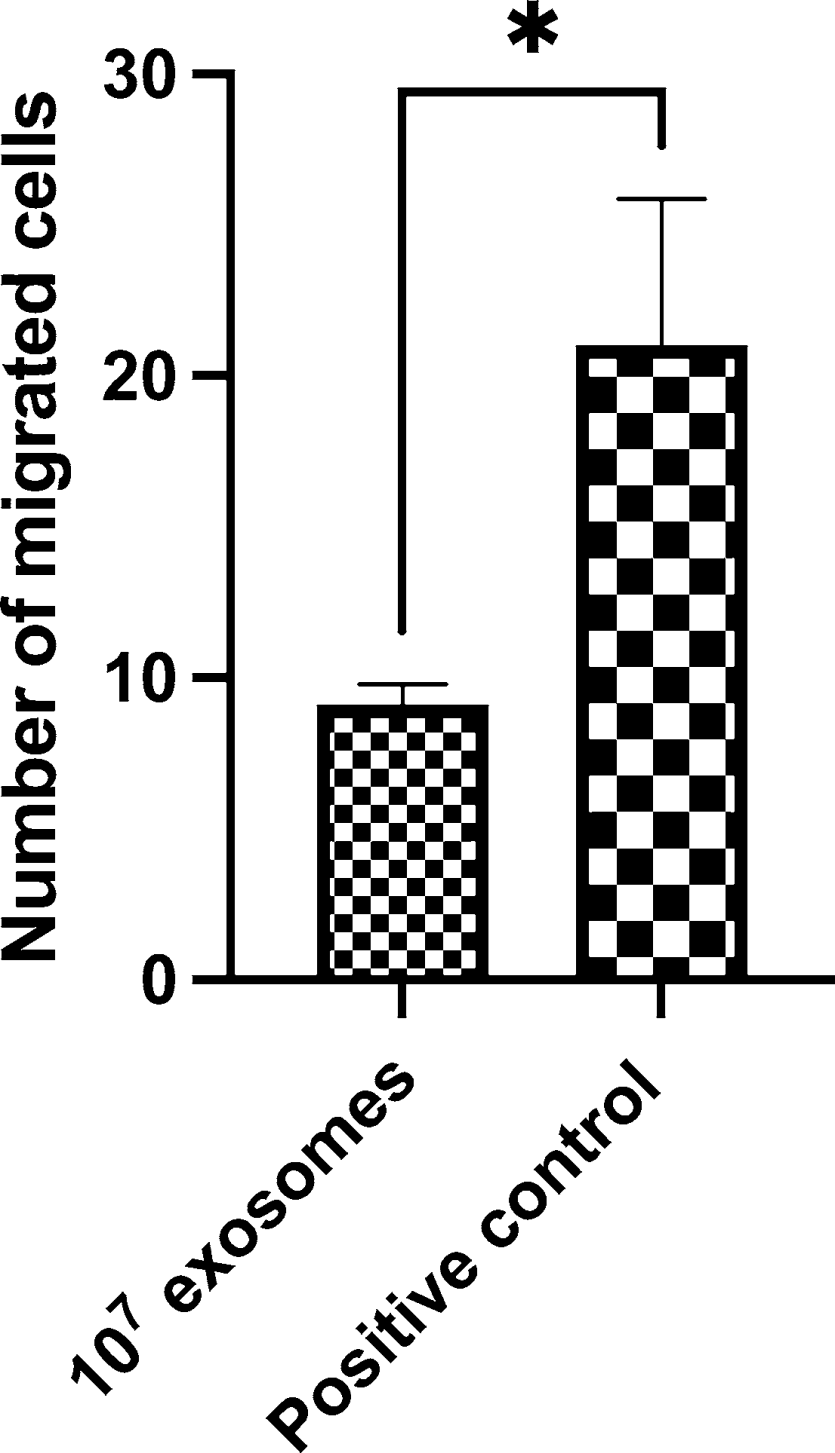
In vitro effects of LPS-depleted porcine milk exosomes (MEx) on porcine monocytes chemotaxis. MEx (10^7^) and medium (positive control) pre-treated cells were both activated with the chemoattractant Zymosan Activated Serum (ZAS) in the presence or absence of MEx, respectively for 2 h. Data are means ± SEM of five experiments. Significance was declared for *P* ≤ 0.05 (*).

The chemotactic activity was measured after activating with the chemoattractant ZAS, the cells either treated with porcine MEx or only medium (positive control). Porcine milk exosomes modulated monocyte chemotaxis (*P* = 0.05), as a decrease in the number of migrated cells was observed when compared with the positive control without exosomes (Fig. 3).

#### Porcine MEx decreased porcine monocytes’ phagocytic capacity, but not their killing capacity

**Figure 4:**
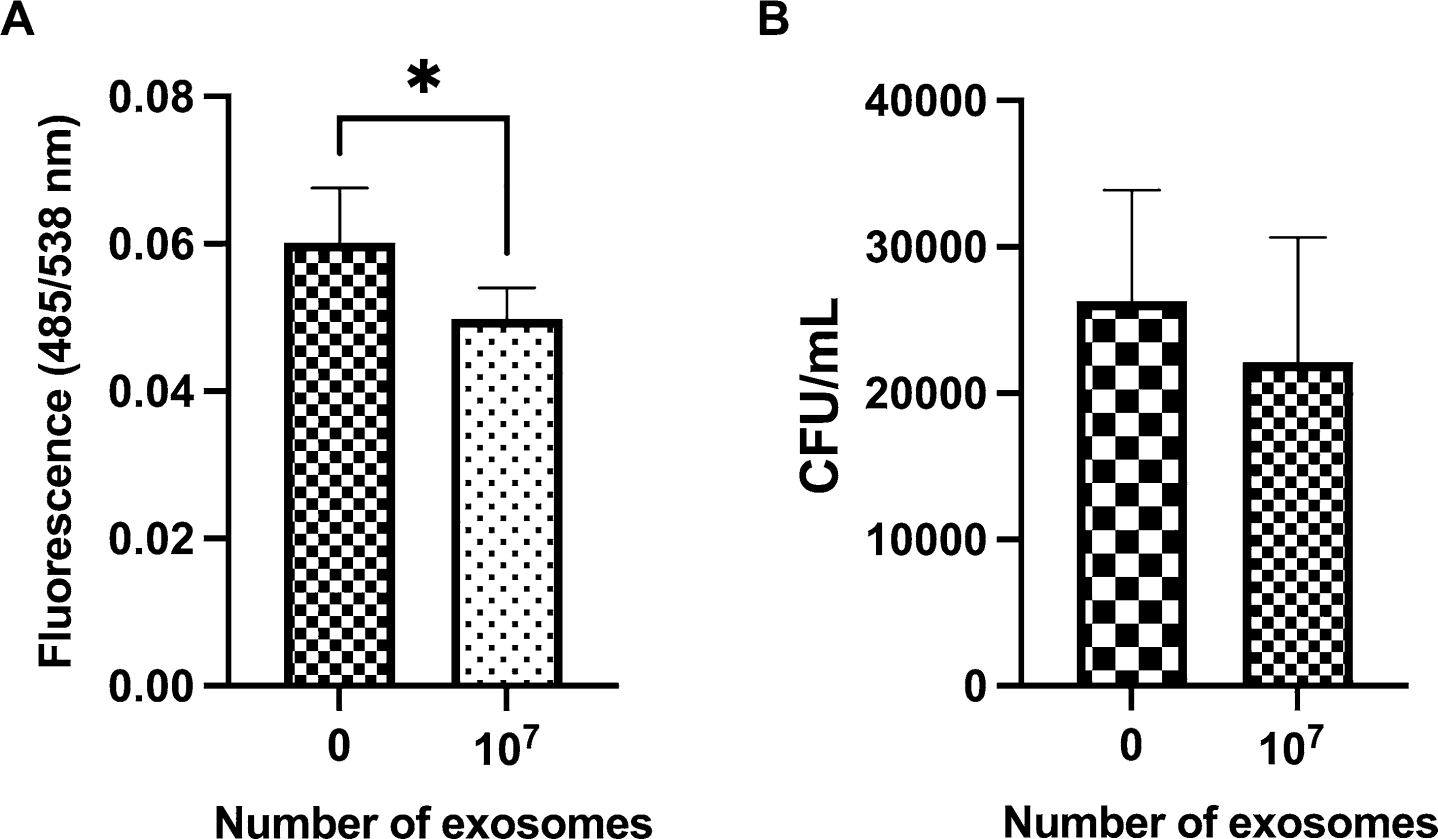
(**A**) Phagocytosis of fluorescein-labelled *Escherichia coli* bioparticles and (**B**) killing capacity of live *E. coli* by porcine monocytes after 22 h incubation with LPS-depleted porcine milk exosomes (MEx) (10^7^). Cells treated with only medium (no exosomes) were considered as control. The results are expressed as fluorescence intensity (485/538 nm) and as colony-forming units/mL (CFU/mL), respectively. Data are means ± SEM of seven and five experiments for phagocytosis and killing capacity, respectively. Significance was declared for *P* ≤ 0.05 (*).

The capacity of monocytes to phagocyte (Fig. 4A) *Escherichia coli* bioparticles when treated with MEx (10^7^) was decreased (*P* = 0.02) compared to the control. However, no effects were observed in their capacity to kill live *E. coli* (Fig. 4B).

#### Porcine MEx modulated porcine monocytes’ ROS production

**Figure 5:**
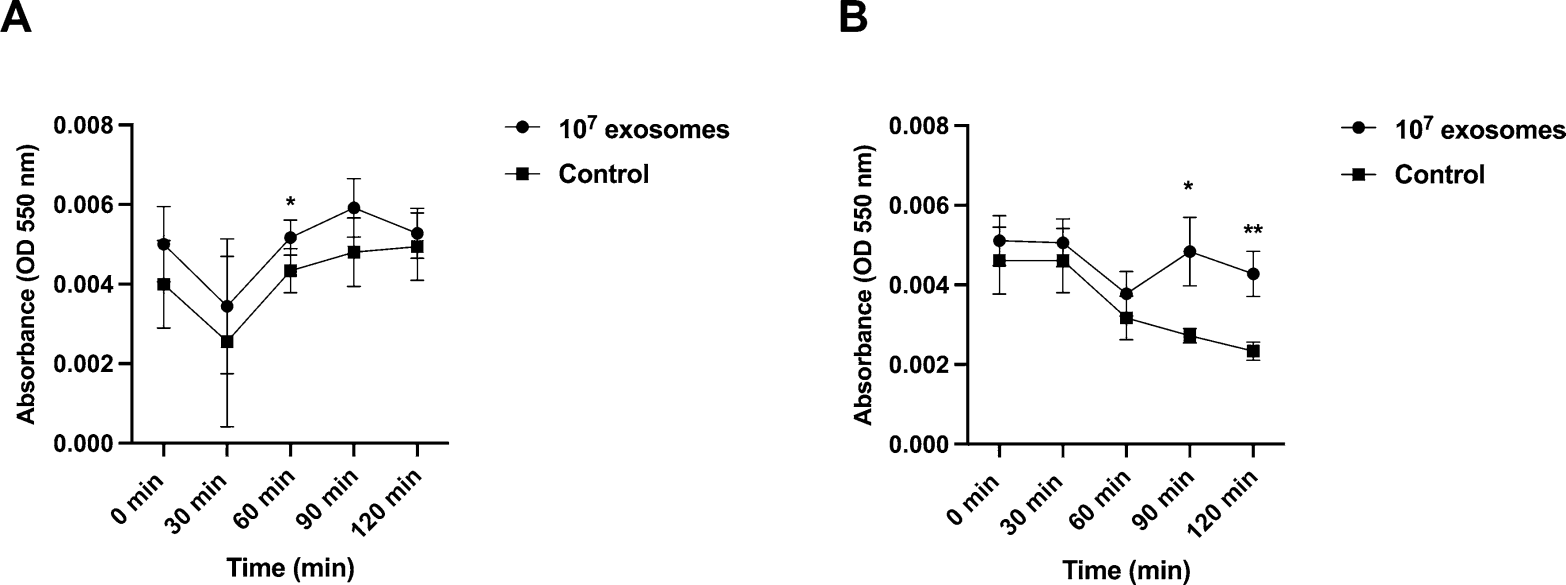
In vitro effects of LPS-depleted porcine milk exosomes (MEx) on porcine monocytes’ extracellular superoxide anion generation (O_2_^−^) after the addition of cytochrome C, under (**A**) non-inflammatory conditions or (**B**) proinflammatory conditions [phorbol myristate acetate (PMA) challenge]. Cells treated with only medium (no exosomes) were considered as control. The results are expressed as absorbance values (OD 550 nm). Data are means ± SEM of six experiments. Significance was declared at *P* ≤ 0.05 (*) and *P* ≤ 0.01 (**). OD, optical density.

The monocytes’ extracellular O_2_^−^ production under non-inflammatory and proinflammatory conditions was also evaluated. Cells co-cultured with porcine LPS-depleted milk exosomes under non-inflammatory conditions showed an increase in ROS production at 60 min (*P* = 0.04) (Fig. 5A) as compared to control. Co-incubation with MEx also affected the ROS production of monocytes after inducing a proinflammatory challenge with PMA; in detail, an increase after 90 min (*P* = 0.03) and 120 min (*P* < 0.01) was detected (Fig. 5B) when compared to the control.

## Discussion

Exosomes are important mediators of intercellular communication. They fulfil this critical role by transferring their cargo of regulatory molecules (nucleic acids, proteins, lipids and metabolites) to recipient cells, altering their biological response, including balancing of the immune response^1^. Exosomes’ immune regulation is thought to be mainly due to: (1) the transfer and presentation of antigens, (2) DNA transfer-inducing cGAS-STING signaling pathway–immune pathway that triggers the expression of inflammatory genes after sensing cytosolic DNA-, (3) miRNA-mediated gene expression regulation, (4) induction of different signaling pathways by exosome surface ligands^1^, and (5) transport of soluble mediators such as cytokines and chemokines^26^.

The contribution of MEx to intercellular communication and regulation of cellular processes remains still undisclosed. To the best of our knowledge, the present study reports for the first time the in vitro uptake of sows’ MEx by porcine monocytes (CD14+), and these exosomes’ ability in modulating monocytes’ immune activities, including chemotaxis, phagocytosis and ROS production. Our main findings were that porcine monocytes internalized porcine MEx, and MEx modulated in vitro the cells’ chemotaxis, phagocytosis, and ROS production, the latter under both, non-inflammatory and proinflammatory conditions.

In the initial phase of our study, we characterized LPS-depleted porcine MEx, revealing their size consistent with the size range previously reported for exosomes (30–200 nm)^3^, and with the observed average size (152 nm) of porcine MEx from different lactation stages^27^. Their concentration also aligned to that obtained previously in porcine milk (2.4 × 10^11^ particles/mL)^27^. TEM images displayed typical exosomal morphology, including round and cup-shaped structures—the latter being related to the drying process for imaging analysis ^5^—and confirmed their lipid bilayer. Immunoblotting verified the presence of the exosome marker protein TSG-101 (tumour susceptibility gene 101), a cytosolic protein involved in the origin and biogenesis process of exosomes^1^, commonly used for defining bovine MEx^28, 29^ and, more recently, used as a main marker for porcine MEx^27^. Moreover, in the latter study, proteomics revealed additional exosome marker proteins, such as CD9 and CD63, in porcine MEx isolated using our method. Altogether, these results reinforce the validity of our isolation approach and the use of the isolated MEx in the further studies.

The second part of the study evaluated the in vitro immunomodulatory activity of MEx on porcine monocytes functions. In a first step, we examined if monocytes take up MEx. Our results revealed that indeed these nanovesicles can be internalized by the cells and that they were located mainly in their cytoplasm near the nucleus, as seen with fluorescence microscopy. These results agree with those of human^17^ and bovine MEx^18^, which can also be taken up by human and murine macrophages^19^*.* In a second step, to rule out any potential toxic effects of porcine MEx and determine their subtoxic working concentration, we evaluated the apoptosis and viability of porcine monocytes. MEx did not affect the monocytes spontaneous apoptosis and viability during the time. These results are consistent with previous studies on human PBMC, where exosomes derived from mesenchymal stromal cells and breast milk did not affect the viability of the cells^30, 31^. Moreover, when co-incubated with other cellular models, such as the porcine intestinal epithelial cells IPEC-J2, MEx have not shown detrimental effects in vitro on the viability when measured with the MTT assay, enhancing their proliferation^23^. However, our results differ from the anti-apoptotic and protective effects that porcine MEx have produced on LPS-treated intestinal epithelial cells, by attenuating their apoptosis and enhancing their viability^32^, suggesting that exosomes do have pleiotropic functions that may depend on the type of cell that produces the exosomes, of the cargo they carry and transfer, and of the recipient cells.

For all the following experiments, a total of 10^7^ exosomes, with a ratio of 200 exosomes/cell and an incubation time of 22 h, were selected, as no toxic effects were observed in our preliminary experiments. This concentration was based on a preliminary study^33^ that investigated the immunomodulatory effects after the internalizing of the exosomes on rhesus macaques PBMC and CD4+ T cells, using a similar range of 10^6^–10^8^ exosomes. Moreover, we decided to select the closest exosome concentration to that found physiologically in porcine milk that could still be used in the in vitro assays.

Porcine MEx decreased monocyte chemotaxis by reducing their migration towards ZAS. To the best of the authors’ knowledge, this is the first study reporting the MEx effects on this activity on porcine immune cells. Proteomic analysis of MEx has confirmed the presence of exosome proteins involved in the migration and cell movement, as pathways such as the chemokine signalling and leukocyte transendothelial migration were found to be enriched^22^. However, exosomes’ effects on chemotaxis likely depend on the source of the exosomes, and the type of the delivered cargo since other contradictory effects of exosomes on chemotaxis have been previously reported^34^.

The monocytes’ phagocytic capacity was decreased by porcine MEx but did not affect their killing capacity. These results could be explained by the immunosuppressive effects already attributed to MEx^35^ and their miRNA cargo^36^ in downregulating murine macrophages phagocytosis via the suppression of nuclear factor-κB (NF-κB)^37^. Proteomic studies of bovine MEx also demonstrated that exosomal proteins were involved in Fcγ receptor-mediated phagocytosis, suggesting that MEx can modulate this inflammatory function^22^. However, the current information on the effects of MEx on phagocytosis is limited, and contradictory, as other studies reported an effect of upregulating phagocytosis^38^.

Our findings show that porcine MEx increased the production of extracellular superoxide anion under non-inflammatory conditions at 60 min in a transitory manner; and in proinflammatory conditions after 90 min of PMA challenge, suggesting that they might play an important role in boosting monocytes’ response, specially in an inflammatory setting. Similar effects have been previously reported in other models, where different sources of exosomes and cellular targets were used, demonstrating that eosinophil-derived exosomes increased the ROS production in patients with asthma^39^. In contrast, macrophage-derived exosomes induced the production of ROS in injured neurons^40^. The molecules necessary for ROS production like NADPH oxidase^41^ and cytochrome P450^42^ were also found in platelet-derived exosomes and plasma exosomes, respectively. However, recent works have instead pointed towards an antioxidant and protective role of exosomes against oxidative stress^43^. For example, bovine MEx inhibit ROS production, increase activities of the antioxidant enzymes SOD and GPX^44^, and suppress murine macrophages ROS production under hypoxic conditions^45^.

## Conclusions

The results of this study demonstrated that LPS-depleted porcine MEx modulate some immune functions of porcine monocytes in vitro. Specifically, MEx decreased monocytes’ phagocytosis and chemotaxis, while increasing their ROS production under non-inflammatory and proinflammatory conditions, suggesting they can exert pleiotropic functions on the cells, as both immunosuppressive and/ or immune-enhancing effects were observed. To the best of our knowledge, we also demonstrated for the first time that monocytes take up MEx in vitro, which could potentially explain the way exosomes exert their immunomodulation. However, our study does not provide evidence on the exact molecular mechanisms underlying such effects. Their elucidation using OMIC technologies like transcriptomics and proteomics would help us to better understand the biological significance in experimental in vivo systems. Moreover, to further elucidate the underlying molecular mechanisms of exosome uptake, it would also be of great interest to perform further in vitro experiments using antibodies to block ligand/receptor interactions or chemical inhibitors of phagocytosis and/or endocytosis (known mechanisms used by cells for exosome internalization).Finally, our results also suggest a potentially critical role of porcine MEx in the sow-to-piglet transmission of regulatory molecules, immunomodulation, and ultimately in the development of the suckling piglet immune system.

## Methods

### Ethics statement

No living animal was used to collect blood samples. The procedures for the blood collection were carried out during routine slaughtering procedures, during the exsanguinating phase. Milk for isolating exosomes was collected at the Teaching and Research Farm Frankenforst of the University of Bonn. This farm holds a permit according to the statutory provisions of the European and German animal welfare law (Art 11, Para 1, Clause 1aTierschG) for breeding and keeping farm animals (cattle, sheep, pigs, chicken, and quail). The milk samples were non-invasively obtained during the naturally occurring milk-let down reflex when the piglets were suckled, without using oxytocin injections. For these reasons, following both German and Italian current legislations, which reflect the EU current legislation, the experimental protocols were not required to be submitted to the named institutional Ethical committee.

### Purification and characterization of porcine MEx

#### Purification of MEx from sows

Exosomes were purified through differential ultracentrifugation and size exclusion chromatography (SEC), as previously reported for porcine MEx with some minor modifications^27^. Milk from multiparous healthy sows (Teaching and Research Farm Frankenforst, University of Bonn) was collected during natural milk ejection. Briefly, sows’ skimmed milk (7.5 mL) was centrifuged at 10,000 g for 30 min at 4 °C to remove the remaining fat, cellular debris and microvesicles. The supernatant was diluted with double-filtered (0.22 µM) sterile PBS to reach a final volume of 12.5 mL and then it was transferred to Ultra-clear quick seal ultracentrifuge tubes (Beckman Coulter, Indianapolis, CA, USA) and ultracentrifuged at 100,000*g* for 1 h at 4 °C, using a fixed rotor (Beckman Coulter TY65 fixed angle rotor, Pasadena, CA, USA). The collected exosomes (2 mL) were thoroughly mixed with the pipette and further purified through SEC, using the qEVOriginal columns from Izon (Izon Science, Oxford, UK), following the manufacturer’s instructions. After the void volume (3 mL), 4 fractions of 500 μL each were collected. The fractions expected to contain the exosomes were pooled and depleted from lipopolysaccharides (LPS) for in vitro studies, using the ToxinEraser Endotoxin Removal Kit (GenScript, Piscataway, NJ, USA) and following the manufacturer’s instructions. The quality of the purification was assessed by NTA, TEM and identification of exosome marker protein by western blotting. The purified LPS-depleted exosomes were stored at − 80 °C until use.

#### Nanoparticle tracking analysis (NTA)

The Nanoparticle Tracking Analysis (NTA) was conducted using a Nanosight NTA 3.3 (Amesbury, United Kingdom) instrument as per the manufacturer’s instructions. The MEx were diluted in double-filtered PBS (1:50), loaded into the chip and the particles were visualized and analyzed with the NTA 3.3 Dev Build 3.3.301 software. For the analysis, the instrument was set up to operate at 22 °C, with a syringe pump speed of 30 arbitrary units (AU) and for each sample, 5 videos of 60 s each were recorded. Results (mean of 5 measurements) are expressed as exosome size (nm) and concentration (particles/mL).

#### Transmission electron microscopy (TEM)

MEx (2.5 µL) were applied to glow‐discharged carbon‐coated formvar copper grids, negatively stained with 2% uranyl acetate, air-dried for 10 min and observed in an FEI Talos 120 kV transmission electron microscope (FEI Company, Netherlands). Images of exosomes were acquired by a 4 k × 4 K Ceta CMOS camera.

#### Identification of exosome marker protein by western blotting

The milk exosome protein concentration was first determined with the Pierce Bicinchoninic acid (BCA) protein assay kit (Thermo Fisher Scientific, Rockford, IL, USA), following the manufacturer’s instructions. Exosomal proteins (2 $\upmu$g) of fractions before and after removal of LPS from exosomes were loaded on sodium dodecyl sulphate–polyacrylamide gel electrophoresis (SDS-PAGE) gel and Western blotted on nitrocellulose membrane, using Trans-Blot Turbo Midi 0.2 µm Nitrocellulose Transfer Packs (Bio-Rad Laboratories, Hercules, CA, USA), and the Trans-Blot Turbo Transfer System (Bio-Rad Laboratories). The membranes were blocked for 1 h with ROTI®Block 1X (Carl Roth, GmbH Co.KG, Schoemperlen, Germany) and incubated with the primary antibody rabbit anti-human TSG-101 (1:2000) (ab225877, Abcam, Cambridge, UK)—a known exosome marker^27–29^—for 2 h at room temperature, and then with the secondary antibody polyclonal anti-rabbit peroxidase (1:3000) (Vector Laboratory, Inc.30, Burlingame, CA, USA) for 1 h at room temperature. The immunodetection of the reactive bands was performed using the Immobilion Western chemiluminescent HRP substrate (Millipore Corporation, Billerica MA, WA, USA).

### Characterization of porcine milk exosomes’ immunomodulatory effects on porcine monocytes

#### Purification of porcine monocytes

Peripheral blood (100 mL) from twenty 60–100 kg healthy pigs (TOPIGS) was collected during routine slaughtering procedures in sterile flasks containing 0.2% of EDTA (Sigma-Aldrich, St. Louis, MO, USA) as an anticoagulant. PBMC were isolated first through Ficoll density gradient centrifugation, as previously described for bovine blood^46^. Briefly, blood was first centrifuged at 1260 g for 30 min at 18 °C to obtain the buffy coat. The buffy coat was diluted 1:5 in cold sterile Dulbecco’s PBS without Ca^2+^ and Mg^2+^ + 2 mM EDTA (Sigma-Aldrich) and carefully layered onto 3 mL of Ficoll-Paque Plus (1.077 g/mL) (GE Healthcare Bio-Sciences AB, Uppsala, Sweden). Cells were then centrifuged at 1700 g (without brakes) for 30 min at 4 °C to obtain the PBMC ring. PBMC were collected at the interface, washed twice with cold sterile PBS without Ca^2+^ and Mg^2+^ + 2 mM EDTA, centrifuged at 500 g for 7 min at 4 °C and incubated with Red Blood Cell Lysis (Roche Diagnostics GmbH, Mannheim, Germany) buffer for 3 min at room temperature to remove the red blood cells. Three consequent washes with cold sterile PBS + 2 mM EDTA were performed to remove contaminant platelets. CD14+ monocytes were further purified from PBMC through magnetic-activated cell sorting technique (MACS), using CD14 MicroBeads, LS (large size) columns and 30 mm pre-separation filters (Miltenyi-Biotech, Bergisch Gladbach, Germany), as previously described for bovine samples^47^, and following the manufacturer’s instructions. Monocytes were counted using an automatic cell counter (TC20™, BioRad), and cells were resuspended in complete medium Roswell Park Memorial Institute 1640 medium (RPMI) with l-glutamine + 25 mM HEPES + 1% P/S + 1% non-essential amino acids and 10% exosome-depleted Fetal Bovine Serum (FBS), purchased from Sigma-Aldrich.

#### Exosome uptake assay

To evaluate if porcine monocytes could internalize porcine MEx, monocytes of healthy animals (four biological replicates) were treated with LPS-depleted MEx and visualized using fluorescence microscopy. First, 10^8^ LPS-depleted exosomes (ratio of 200 exosomes/cell) were labelled with the PKH26 Red Fluorescent Cell Linker Mini (Sigma-Aldrich), following the manufacturer’s instructions with minor modifications. Briefly, LPS-depleted MEx or PBS (negative control) were mixed with 250 μL of Diluent C and then rapidly added to a PKH26 dye solution in diluent C (0.04 × 10^–6^ final concentration), which was prepared immediately before staining. The exosomes were incubated with periodic mixing for 5 min in dark, and then 10% of exosome depleted serum was added to stop the staining and allow the binding of excess dye. The excess of unincorporated dye was further removed with the Exosome Spin Columns (MW3000) (Invitrogen, Waltham, MA, USA), following the manufacturer’s instructions. Before cell seeding, the sterile 4-well Nunc Lab-Tek II Chamber Slides w/Cover RS Glass Slide (Thermo Fisher Scientific, Waltham, MA, USA) were treated with 100 μL of Poly-d-lysine (50 μg/mL) (Sigma-Aldrich) for 2 h, to enhance the cells’ adherence, and washed with pyrogen-free water. Then, 5 × 10^5^ monocytes, purified from 4 animals, were seeded and co-incubated with 10^8^ PKH26-labeled exosomes (150 μL) or PBS as a negative control for 22 h at 39 °C in a humidified atmosphere and 5% CO_2_. After the incubation, cells were fixed with 4% paraformaldehyde (PFA) for 30 min at room temperature and the nuclei were stained with Hoechst 33,342 (Sigma-Aldrich) (1 μg/mL) for 15 min. Finally, a drop of Invitrogen™ProLong™ Diamond Antifade Mountant (Thermo Fisher Scientific) was added to the slides and the cells were visualized using a fluorescence microscope (Eclipse E600; Nikon). The images were then analyzed with the ImageJ/Fiji software. The percentage of PKH26-exosome positive cells was determined by calculating the ratio between the cells positive for the red fluorescent dye and the total number of cells observed in each field, multiplied by 100. Results are expressed as the mean percentage of three different fields. 

#### Viability assay

The viability assay was performed to assess the potential cytotoxicity of LPS-depleted MEx on porcine monocytes by using the Cell proliferation kit I (MTT) (Roche Diagnostics) as already reported for bovine monocytes^47^. A total amount of 1 × 10^5^ cells (25 µL) per well, was seeded in duplicate in sterile 96-well plates (Becton Dickinson and Company, Franklin Lakes, NJ, USA). The plates were incubated for 12 h and 22 h at 39 °C in a humidified atmosphere and 5% CO_2_ with increasing numbers (10^3^, 10^5^, 10^7^, and 10^8^) of LPS-depleted MEx (25 µL) or with the medium as control (no exosomes). The study was carried out on an average of five biological replicates. To measure the cells’ viability, 10 µL of the MTT labeling reagent were added to each well and incubated at 39 °C for 4 h, following the manufacturer’s instructions. The formazan crystals were solubilized by adding 100 µL of solubilizing buffer and incubating the plates overnight at 39 °C. The absorbance was read at 550 nm with a LabSystem Multiskan plate reader Spectrophotometer (LabX, Midland, Canada).

#### Apoptosis assay

To evaluate whether MEx could affect porcine monocytes’ apoptosis, the enzymatic activity of Caspase-3/7 was measured. Briefly, 5 × 10^4^ cells (12.5 µL) were seeded in duplicate in sterile 384-well black plates (Corning Inc., Kennebunk, ME, USA), as previously described for bovine samples^46^. The study was carried out on an average of five biological replicates. The cells were incubated for 12 h and 22 h at 39 °C in a humidified atmosphere and 5% CO_2_ with increasing numbers (10^3^, 10^5^,10^7^, and 10^8^) of LPS-depleted porcine MEx (12.5 µL) or the medium as control (no exosomes). The apoptosis assay was carried out using the Apo-ONE® reagent Homogeneous Caspase-3/7 kit (Promega, Madison, WI, USA), following the manufacturer’s instructions. The fluorescence of 485/538 nm (absorbance/emission) was measured with a fluorescence plate reader Fluoroscan Ascent (Thermo Fisher Scientific). 

#### Chemotaxis assay

The monocytes’ chemotaxis towards zymosan activated serum (ZAS)—a known chemoattractant—was measured as previously reported, with some minor changes^47^. The study was carried out on an average of five biological replicates. Briefly, ZAS was prepared as previously described^48^, and 50 µl of purified monocytes (1 × 10^5^) were seeded in duplicate in the upper chamber of a sterile 24-well Transwell migration plates (Corning Inc.), equipped with an 8 µm pore size membrane. Cells were then pretreated with 2 × 10^7^ LPS-depleted MEx (a ratio of 200 exosomes/cell), in the absence of a chemoattractant, for 22 h at 39 °C and 5% CO_2_. After the incubation, ZAS (3 mg/mL) was added as chemoattractant in the lower chamber, and the cells were incubated either in the presence of MEx or only medium (RMPI-1640 with 1% of exosome-depleted FBS) as positive control (no exosomes) again for 2 h at 39 °C and 5% CO_2_. Finally, non-migrated cells were gently removed with a swab moistened with PBS, and migrated cells were stained with Diff-Quick (Sigma-Aldrich) and counted in 10 different fields, using light microscopy (inverted microscope). 

#### Phagocytosis assay

The phagocytosis assay was carried out by measuring the fluorescence of fluorescein-labelled *Escherichia coli (E. coli)* K-12 strain bioparticles (Invitrogen) as previously performed^49^. The study was carried out on an average of seven biological replicates. Opsonisation of fluorescein-labelled *E. coli* bioparticles (K-12 strain) was performed by incubating 80 µL of bacteria suspension (5 × 10^6^ *E.coli*/µL) with 20% of exosome-depleted FBS (20 µL) for 30 min at 39 °C. The suspension was centrifuged at 800×*g* for 15 min and suspended in PBS. A total of 3 × 10^5^ monocytes (100 µL) were seeded in duplicate in 96-well plates and treated with 100 µL of LPS-depleted MEx (ratio of 200 exosomes/cell) or with the medium as control (no exosomes). Cells were then incubated at 39 °C and 5% CO_2_ for 22 h. The cells were washed with PBS and fluorescein-labeled *E. coli* bioparticles with a ratio of 45 particles/cell were added and co-incubated for 2 h at 39 °C. Cells were washed with PBS to remove non-internalized bioparticles and incubated with 50 µL trypan blue 0.4% for 1 min at room temperature to remove and quench the fluorescence of the non-internalized bacteria, respectively. After removal of the trypan blue, the fluorescence (485/538 nm) was measured using the microplate reader Fluoroscan Ascent (Thermo Fisher Scientific). 

#### Killing capability assay

The intracellular bacterial killing capacity was determined as reported previously^50^. The *E. coli* American Type Culture Collection (ATCC) 25,922 (strain Seattle 1946; LCG Standards Ltd., Teddington, UK) were opsonized with 20% exosome depleted FBS (20 µL), incubated at 37 °C for 30 min. The bacteria were washed twice by centrifugation at 1500 g for 10 min at 4 °C and suspended with PBS. A total of 3 × 10^5^ monocytes (100 µL) was seeded in duplicate in cryogenic vials. The study was carried out on an average of five biological replicates. Cells were then treated with 6 × 10^7^ LPS-depleted MEx (100 µL), in a ratio of 200 exosomes/cell, or with the medium as control (no exosomes) and were incubated for 22 h at 39 °C and 5% CO_2_. After the incubation, cells were incubated for 1 h at 39 °C and 5% CO_2_ with 1 × 10^7^ opsonized live *E. coli*. The unbound bacteria were removed by centrifugation and by further treating the cells with 100 µg/mL of gentamicin for 1 h. Gentamicin was eliminated by washing the cells with PBS and centrifuging at 110×*g* for 5 min. Finally, cells were lysed with 0.5% Triton x-100 (Sigma-Aldrich) on ice for 10 min, and after overnight incubation at 37 °C, the colonies forming units (CFU) of surviving bacteria were counted on MacConkey agar plates. Results were then expressed as CFU/mL.

#### Reactive oxygen species (ROS) production assay

As previously described, the production of extracellular superoxide anions (O_2_^−^) was determined with the cytochrome C reduction assay^49^. The study was carried out on an average of seven biological replicates. A total of 1 × 10^5^ monocytes (50 μL) was seeded in complete medium without phenol red in triplicate in 96-well sterile plates and co-incubated for 22 h at 39 °C + 5% CO_2_ with 2 × 10^7^ exosomes (50 μL) in a ratio of 200 exosomes/cell. After the incubation period, the production of O_2_^−^ was measured under non-inflammatory and proinflammatory conditions when challenged with phorbol myristate acetate (PMA). In the non-inflammatory conditions, 10 μL of Cytochrome C from the equine heart (1 mM), (Sigma-Aldrich) were added; while to mimic a proinflammatory challenge 10 μL of Cytochrome C and 2 μL PMA (2.5 μg/mL final concentration; Sigma-Aldrich) were added. Medium without phenol red was added to all wells to have a final volume of 200 μL. The absorbance was measured every 30 min for 4 h at 550 nm with a LabSystem Multiskan plate reader spectrophotometer (LabX).

### Statistical analyses

Statistical analyses were performed in GraphPad Prism 8.0.2. Data normality was assessed with the Shapiro Wilk test. Repeated measures one-way ANOVA and Tukey’s multiple comparison tests were used for normally distributed samples from viability and apoptosis at 22 h assays. In contrast, a Friedman test and Dunn’s multiple comparisons tests were applied for apoptosis at 12 h. Paired t-tests were used for phagocytosis, killing capacity, chemotaxis, ROS production and exosome uptake assays. Statistical differences were accepted at *P* ≤ 0.05.

### Supplementary Information

Supplementary Information 1.

Supplementary Video 1.

## Data Availability

The datasets generated during and/or analyzed during the current study are available from the corresponding author on reasonable request.
