# Supplementary material for: Porcine milk exosomes modulate the immune functions of CD14+ monocytes in vitro
Source: Sci Rep. 2023 Dec 5;13:21447. doi: 10.1038/s41598-023-48376-y (PMC10698175; doi:10.1038/s41598-023-48376-y)
Supplement: Supplementary file 1 — Supplementary Information 1. [file 41598_2023_48376_MOESM1_ESM.pdf]

Supplementary information

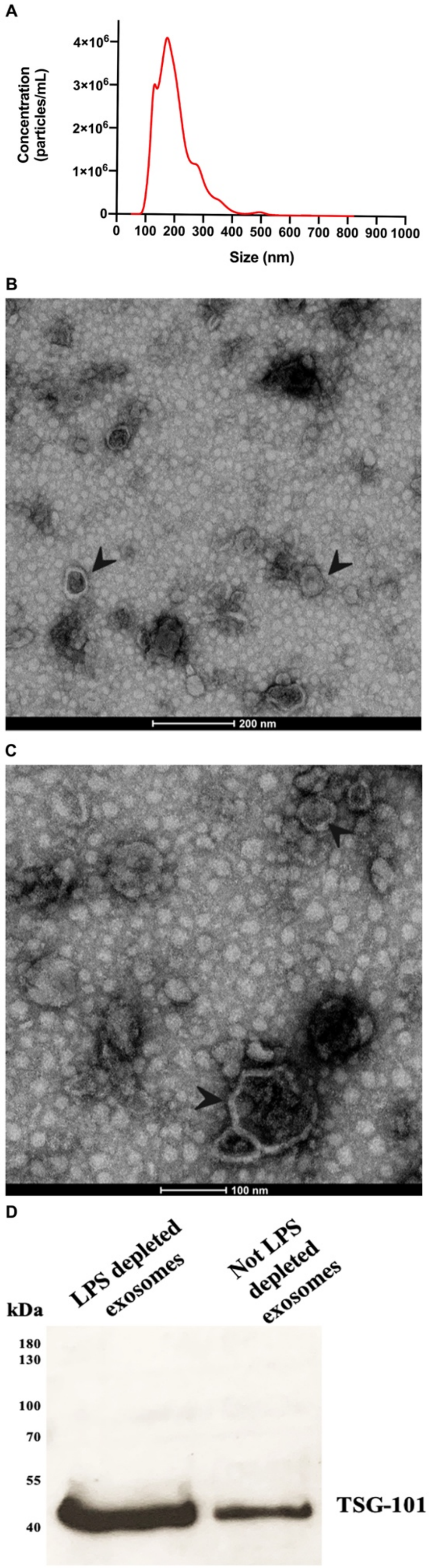

Supplementary Figure 1. Characterization of LPS-depleted porcine milk exosomes (MEx) purified by ultracentrifugation coupled with Size Exclusion Chromatography (SEC). (A) Nanoparticle Tracking Analysis (NTA) of LPS-depleted porcine MEx. The plot of NTA analysis shows the size (nm) on the x-axis and the concentration (particles/mL) on the y-axis. The size of the exosomes was 156 ± 8.9 nm. Transmission Electron Microscopy (TEM) micrographs showed that porcine MEx have a characteristic (B) round and/or cup shape and are (C) limited by a lipid bilayer, indicated by the black arrows. Scale bars: 100 and 200 nm, respectively. (D) Representative western blotting analysis of the exosome marker TSG-101 in porcine milk exosomes. TSG-101 (44 kDa) was detected in both LPS-depleted (first lane) and non-LPS-depleted porcine milk exosomes, confirming the isolation of milk exosomes.

Supplementary Video1. Porcine LPS-depleted milk exosomes (MEx) visualization through Nanoparticle Tracking Analysis (NTA).
